# Primary Immunodeficiency in a 74-Year-Old Male With Chronic Productive Cough: A Rare Case of Common Variable Immunodeficiency

**DOI:** 10.7759/cureus.20273

**Published:** 2021-12-08

**Authors:** Ahmed Elkhapery, Sravani Lokineni, Zeinab Abdalla

**Affiliations:** 1 Internal Medicine, Rochester Regional Health, Rochester, USA; 2 College of Medicine, University of Sharjah, Sharjah, ARE

**Keywords:** intravenous immunoglobulins (ivig), copd: chronic obstructive pulmonary disease, chronic refractory cough, non-cf bronchiectasis, common variable immunodeficiency (cvid)

## Abstract

Common variable immunodeficiency (CVID) is the most common symptomatic primary immunodeficiency. It presents with variable degrees of immunodeficiency resulting in a variety of clinical presentations and complications. This report describes the case of newly diagnosed CVID in a 74-year-old man with no history of recurrent infections or hospitalizations. He presented with chronic productive cough, wheezing, shortness of breath and fatigue. Physical examination showed bilateral rhonchi and scattered wheezes. Pulmonary function tests showed moderate obstructive defect with partial reversibility and decreased diffusion lung capacity for carbon monoxide (DLCO). Chest computed tomography (CT) showed bilateral lower lobe peribronchial thickening and mildly enlarged lymph nodes in the mediastinum and upper abdomen. Bronchoscopy with alveolar lavage was done and respiratory samples grew *Moraxella*. He had negative acid fast bacillus stain and negative tuberculosis and fungal cultures. He received a course of antibiotics resulting in brief improvement in symptoms followed by another exacerbation. Repeat sputum cultures grew *Pseudomonas*. Further testing showed severely depressed levels of immunoglobulin. His symptoms ultimately improved with immunoglobulin replacement therapy. A broad differential, including CVID, needs to be considered in working up a patient with a chronic productive cough and recurrent lower respiratory tract infection.

## Introduction

Common variable immunodeficiency (CVID) is the most common symptomatic primary immunodeficiency. It is most prevalent in Caucasians and less common in African American and Asian populations [1]. It presents with variable degrees of immunodeficiency resulting in a variety of clinical presentations and complications. It is caused by abnormal differentiation of B cells into plasma cells, resulting in a significantly reduced immunoglobulin production of at least two isotypes, along with other immune system defects [2]. Most patients are diagnosed at 20-40 years of age and it is exceedingly rare to diagnose in older adults, likely partly due to underdiagnosis. There are delays of five to 8.9 years reported in various cohorts [3-5]. This is especially important because diagnostic delay is associated with a statistically significant increase in mortality [6]. Here we present a case of newly diagnosed CVID in a 74-year-old male who presented with a chronic productive cough.

## Case presentation

A 74-year-old male with a history of coronary artery disease, stent placement, and hypertension was referred to pulmonology for a chronic productive cough. He had no history of recurrent infections or hospitalizations. He reported eight months of persistent productive cough, particularly at night, with wheezing/shortness of breath. He was treated with antibiotics and steroids on two occasions, with brief improvement followed by recurrence of symptoms each time. He reported decreased appetite, fatigue, and weight loss of 20-25 pounds. He had no prior hospitalizations for pneumonia and had an unremarkable family history. He was a former smoker (9 pack years), quit approximately 40 years ago.

Physical examination showed bilateral rhonchi and scattered wheezes involving all fields bilaterally, a normal cardiovascular exam, and no notable lymphadenopathy, hepatosplenomegaly, or skin abnormalities. Pulmonary function tests (PFTs) showed moderate obstruction with reduction in forced expiratory volume in one second (FEV1)/forced vital capacity (FVC) ratio (69%) and reduction of FEV1 (67% of predicted), with mildly decreased diffusion lung capacity for carbon monoxide (DLCO) (Table 1).

**Table 1:** Pulmonary function testing results, pre- and post- bronchodilator FVC (forced vital capacity), FEV1 (forced expiratory volume in one second), FEF25%–75% (forced expiratory flow at 25% to 75% of FVC), DLCO (diffusion lung capacity for carbon monoxide)

Spirometry		Pre-Bronchodilator			Post-Bronchodilator		
		Actual	Predicted	% Predicted	Actual	% Predicted	% change
FVC	L	3.27	4.62	71	3.78	82	16
FEV1	L	2.25	3.37	67	2.62	78	16
FEV1/FVC	%	69	73	95	69	95	0
FEF25%–75%	L/s	1.51	2.47	61	-	-	
Diffusion							
DLCO		18.57	28.12	66			

**Figure 1:**
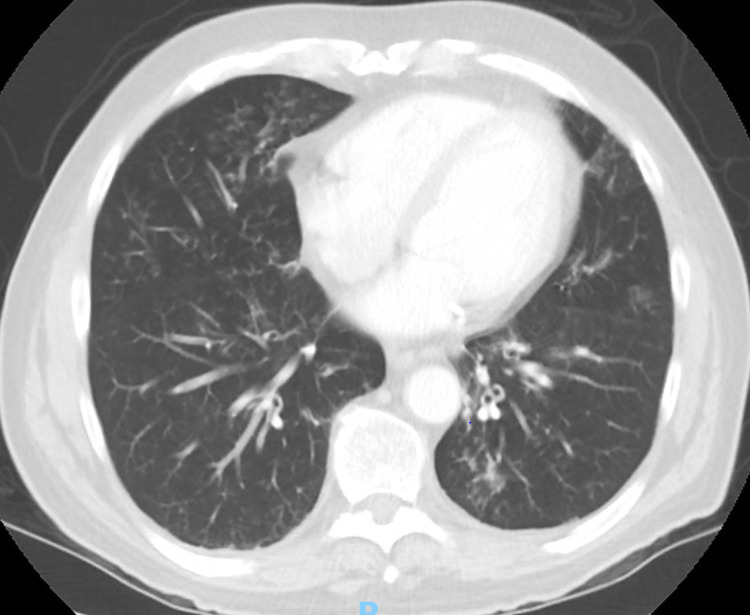
CT Chest with contrast showing mild to moderate peribronchial thickening, multiple foci of mucoid impaction, and ill-defined areas of ground-glass opacity, with tiny clustered nodules/tree-in-bud opacities and right middle lobe, lingular, and bilateral lower lobe predominance.

There was also a reduction of FVC (71% of predicted) and a significant bronchodilator response. As a result of the PFT findings, a steroid inhaler trial was prescribed, which had little benefit. Chest computed tomography (CT) was done, which showed bilateral lower lobe peribronchial thickening and mildly enlarged lymph nodes in the mediastinum and upper abdomen (Figure 1). 

A bronchoscopy with bronchoalveolar lavage was done from which samples grew *Moraxella*, while acid fast bacillus stain, tuberculosis cultures, and fungal cultures were negative. A 10-day course of moxifloxacin was prescribed, which resulted in brief improvement, followed by recurrence of symptoms a few weeks later. The patient’s repeat sputum culture one month later grew *Pseudomonas*, and he was treated with a 10-day course of levofloxacin, which again resulted in brief improvement. Repeat chest CT showed mild progression of disease, with patchy alveolar ground glass opacities and bronchial wall thickening with mucoid impaction (CT scan images).

At this point, workup for immunodeficiency was done, which revealed severely depressed levels of immunoglobulin (IgG, IgM and IgA) (Table 2).

**Table 2:** Specific lab workup

Laboratory Workup	Values	Reference range
IgG	<70	700-1600 mg/dl
IgM	<8	50-300 mg/dl
IgA	<18	70-400 mg/dl
CD4 count	510	485-1376/mm3
CD8 count	467	289-796/mm3
CD19 count	75	111-480/mm3
HIV serology	Negative	Negative
Hepatitis B surface AG	Negative	Negative
Hepatitis B surface AB	Negative	Negative
Hepatitis B Core antibody, total	Negative	Negative
Hepatitis C antibody	Negative	Negative
Cystic fibrosis mutation testing	None detected	
Pneumococcal antibody levels (23 serotypes)	Non-detectable	
Diphtheria IgG antibody	Non-detectable	
Tetanus IgG antibody	Positive (0.05)	>= 0.01 IU/mL
Alpha 1 antitrypsin level	231	100 - 190 mg/dL
Bone marrow biopsy	Negative for malignancy	

After one month of weekly subcutaneous IgG replacement therapy, the patient’s IgG levels reached 445, with decreased coughing/wheezing. Follow-up CT chest showed near-complete resolution of ground-glass opacities. Repeat PFT showed normal spirometry with persistent mild reduction in DLCO.

Further investigations were done to rule out lymphoma as a secondary cause of hypogammaglobinemia, including CT abdomen looking for lymphadenopathy, and bone marrow biopsy. Bone marrow biopsy results showed hypercellular marrow, as well as granulomas without definite necrosis and negative stains for mycobacteria and fungi. There was focal lymphocytic hyperplasia. Immunostains and flow cytometry showed no evidence of lymphoma.

## Discussion

CVID is an acquired immunodeficiency where there is an abnormal differentiation of B cells into plasma cells, resulting in reduced immunoglobulin production of at least two isotypes. The incidence of CVID is estimated to be between 1:20,000 and 1:50,000 in Caucasians and is less common in patients of Asian or African American origin. Most patients are diagnosed at 20-40 years of age, with a bimodal peak of onset in the first and third decades of life as reported an American cohort of 248 patients [3], while a European cohort of 413 patients showed a mean age of onset of 35.3 years and a median of 33 years [4].

The clinical manifestations and complications of the disease may overlap, but in general, patients present with multiple recurrent respiratory and gastroenteric infections, often accompanied by organ damage. CVID can cause lung dysfunction through recurrent sinopulmonary infections, bronchiectasis, sarcoidosis-like disease, or granulomatous interstitial lung disease [7]. GI disease can manifest as inflammatory bowel disease [1], malabsorption syndrome or bacterial overgrowth, as well as infectious diarrhea caused by pathogens such as *Giardia lamblia*, *Cryptosporidium parvum*, *Cytomegalovirus*, *Salmonella* species, and *Campylobacter jejuni* [8,9]. Data on 2212 patients with CVID were described in a European multicenter retrospective study, with pneumonias occurring in 32% of patients, autoimmunitiy in 29%, enlarged spleen in 26%, bronchiectasis in 23%, GI disease in 9%, multisystem granulomas in 9%, malignancy in 5% and lymphoma in 3%. They showed that enteropathy, autoimmunity, granulomas and splenomegaly often cluster in patients [10].

CVID is often a diagnosis by exclusion, and the associated delays can result in permanent organ damage. The Pan-American Group for Immunodeficiency and the European Society for Immunodeficiencies in 1999 and more recently The International Consensus Document for CVID disorders in 2016 established a diagnostic standard that can delineate the disease and provide a standard definition. It contains three criteria confirmed by qualitative IgGs: Low levels of IgG (defined as two standard deviations below the mean), Poor response to vaccines, and Exclusion of other primary and secondary causes of hypogammaglobinemia. This highlights that symptoms are variable and are not used as diagnostic criteria, while at the same time asymptomatic patients who meet the criteria are considered to have CVID. Causes of acquired hypogammaglobulinemia should always be ruled out during the workup for CVID, including chronic glucocorticoid use, antiepileptic drugs, rituximab therapy, certain lymphomas and leukemias, nephrotic syndrome, and protein-losing enteropathy [11,12]. 

There is often a significant delay between the onset of symptoms and diagnosis, which results in increased morbidity and mortality [6]. A diagnostic delay of four to five years was reported in a European study [10], while a five- to six-year delay was reported in an American cohort [3], and an 8.9 years delay was reported in an Italian cohort [5]. This emphasizes that physicians need to maintain an index of suspicion for immunodeficiencies in patients of all ages.

People with CVID are treated with IgG replacement therapy intravenously or subcutaneously, and many studies have proven that this treatment improves quality of life, ameliorates the severity of CVID, and slows the progression of complications [13]. The earlier this diagnosis is made the better, with each year of diagnostic delay being associated with an increase in the risk of death by 4%, bronchiectasis by 3%, solid tumor by 8%, and enteropathy by 2% [6].

## Conclusions

CVID is the most common primary immunodeficiency and can present at any age. It is likely under-diagnosed, which can result in increased morbidity and mortality. A broad differential, including CVID, needs to be considered in working up patients with chronic cough and recurrent lower respiratory tract infections. This should be done at an early stage to improve patient outcomes.
